# Health messages that engage audiences after the COVID-19 pandemic: content analysis of Chinese posts on social media

**DOI:** 10.3389/fpubh.2025.1533390

**Published:** 2025-03-18

**Authors:** Jing Yu, Yitong Zhao

**Affiliations:** ^1^School of Communication, East China Normal University, Shanghai, China; ^2^School of Politics and International Relations, East China Normal University, Shanghai, China

**Keywords:** audience engagement, content analysis, social media, health message strategies, health communication

## Abstract

**Background:**

After China lifted strict COVID-19 control measures, the winter of 2023 saw widespread outbreaks of emerging infectious diseases, including various strains of influenza, mycoplasma pneumonia, and COVID-19. These diseases have heightened the risk of repeated infections and severe illness, making health communication strategies increasingly important for health promotion. While most studies focus on message dissemination by authorities and experts, the role of patient-generated content, shared by individuals with firsthand health experiences, remains underexplored.

**Methods:**

In this context, social media platforms like Xiaohongshu provide a new avenue for users to share personal health experiences, which have become an important aspect of health communication. This study aims to explore how health communicators can use different communication strategies to effectively engage audiences after the pandemic. By conducting a content analysis of 1,824 posts related to emerging infectious diseases on Xiaohongshu after the COVID-19 pandemic, we examine the relationship between health message strategies and audience engagement.

**Results:**

The results reveal that, in terms of message content, textual strategies such as information-focused language and source credibility cues positively impact audience engagement. In contrast, rich visual content negatively affects engagement. Regarding message style, strategies that include affective, interactive, and cohesive cues in text, as well as the use of warm colors in visuals, positively predict audience engagement.

**Conclusion:**

This study develops an integrated framework for health communicators to effectively use social media to share personal health experiences and engage audiences in collective responses to health crises.

## 1 Introduction

After China lifted strict COVID-19 control measures, the winter of 2023 saw widespread outbreaks of emerging infectious diseases, including various strains of influenza, mycoplasma pneumonia, and COVID-19. These pathogens, characterized by limited immunity duration and a lack of cross-protective immunity, increased the risk of repeated infections and potential severe illness. Acute infectious disease outbreaks pose significant threats to human health, and existing studies have predominantly explored health information dissemination from the perspectives of authorities, experts, or professional agencies to boost public engagement. However, the role of information shared by patients as firsthand witnesses remains underexamined. Patient-generated content, based on personal health experiences and shared on platforms, is highly relatable for the public, attracting audience engagement.

Due to the complexity and unpredictability of the transmission and treatment of emerging infectious diseases, along with the strain on medical resources, patients and their families tend to seek convenient and immediate health information on social media platforms like Xiaohongshu to guide their treatment decisions. These platforms have become crucial for sharing medical information, understanding illnesses, and exchanging personal health experiences. In health-related social media, users' communication is bidirectional. Users are not only seekers of social support but also providers of support by sharing their own experiences and advice. Therefore, patients and their families are willing to share their disease experiences online, particularly regarding treatment processes and various medical aspects, to provide informational support and assist others in taking effective measures.

Xiaohongshu, a multimodal social media platform centered on images, text, and videos, has garnered immense popularity among women and teenagers in China. It serves as a typical “planting grass” platform where users engage in discussions about health and disease, thereby improving health outcomes (1). By sharing health information and personal experiences, Xiaohongshu plays a pivotal role in reducing disease-related uncertainty and fulfilling users' rational needs for health-related knowledge (2). Meanwhile, as a social media platform in China, Xiaohongshu is permeated by the influence of Chinese culture on communication, such as group solidarity, emphasis on comfort, and the reflection of interpersonal understanding and tolerance. In particular, the intimate interactions fostered by a sense of community among acquaintances meet users' emotional needs.

Social media provides new channels for individuals to share their experiences and plays an increasingly important role in promoting the sharing of public health messages. However, not all health messages lead to successful persuasive effects and positive health outcomes (3). Previous research has not adequately explored how health communicators can develop higher-quality and more targeted health information dissemination strategies, which can foster meaningful interactions and encourage positive health behaviors.

This study aims to identify the “active ingredients” of health message strategies to engage audiences. The objective is to develop a comprehensive framework for analyzing the textual content and visual characteristics of these posts, assessing their impact on audience engagement. The findings will offer valuable insights for health communicators regarding effective information dissemination strategies on social media, providing a more nuanced perspective on audience engagement during public health events. Furthermore, this work can draw insights from personal illness narratives to inform treatment processes in health promotion, providing practical guidance for online community managers, healthcare professionals, and health authorities.

## 2 Literature review

### 2.1 Audience engagement

Engagement has been defined as “the willingness to invest in the undertaking of focal interactions with particular engagement objects” (4). Health promotion messages can be strategically designed to enhance audience engagement (5). Audience engagement on social media is multidimensional (6). Social media enables users to interact with information through functions such as likes, shares, and comments (7). Engagement goes beyond the simple exchange of information and is crucial for minimizing uncertainty, anxiety, and distress during crises (8).

We focus on four specific types of engagement on social media: likes, favorites, comments, and shares. These different engagement behaviors represent varying levels of effort (9). Liking indicates a user's approval of the content. With a simple click of the “like” button, users can express their preferences and more intuitively acknowledge online content. Commenting is an active engagement behavior, involving the process of forming opinions and expressing thoughts. This behavior requires more cognitive effort, including thinking and writing (10). On Xiaohongshu, favorites represent users' deep recognition of the content, which is more of a long-term emotional investment. Favoriting increases the likelihood of future viewing (11). Sharing consciously provides content to friends. Sharing often indicates strong user agreement with the shared content, reflecting a higher level of psychological effort (9).

For effective health promotion, it is essential to understand how to meaningfully engage audiences through social media messaging. This study, focusing on both message content and message style, addresses the following research question: How do health message strategies and their sub-dimensions relate to different types of audience engagement on Xiaohongshu?

### 2.2 Health message strategies

Effective public health message strategies ensure that more people can access, understand, and engage with messages during health crises (12). Prior research identifies two critical components of effective public health messaging: message content and message style (13). Guided by this framework, this study examines both the textual and visual aspects of message content and style of posts on Xiaohongshu. Message content provides information through facts, statistics, and other relevant data, fostering logical thinking that helps users make decisions to optimize benefits while minimizing risks and search costs. This informative approach enhances audience engagement. Message style emphasizes the distinctive characteristics of information delivery, activating users' emotional responses and driving engagement. By strategically shaping how information is conveyed, message style fosters perceptions and social interactions that encourage participation (14).

In terms of textual message content, we predict that strategies aimed at reducing uncertainty will attract greater public engagement. Uncertainty Reduction Theory (URT), suggests that people seek reliable, detailed information to ease uncertainty during crises, leading to positive outcomes (15, 16). Firstly, by providing event-based information on disease processes, including diagnosis, prognosis, treatment plans, symptoms, financial aspects, and recovery, uncertainty can be effectively reduced (17). Health messages that focus on these events can promote audience engagement, as users may find the information compelling and be motivated to respond and share it with others (18). Secondly, providing information-focused language cues can also effectively engage audiences. Information-focused language content, characterized by informativeness, is verbally rich and typically involves delivering factual or evidential information. Finally, providing source credibility cues (19), such as the expertise, capability, and credibility indicators of information providers (including scientific evidence and source proofs), can reduce uncertainty and thus enhance audience engagement (20, 21). In summary, we propose three distinct strategies to reduce uncertainty in health crisis communication on social media: 1. providing event-based information on disease processes; 2. providing information-focused language cues; 3. providing source credibility cues.

H1: There are significant predictions of audience engagement by textual message content (1. providing event-based information on disease processes; 2. providing information-focused language cues; 3. providing source credibility cues).

In terms of visual message content, with technological advancements in the combined visual and auditory communication functions, visual message content has gained a strong advantage in engaging audiences, since it can generate more information-rich cues (22). First, more images offer richer visuals, showing various angles, details, and scenes. Especially in health crisis situations, multiple images can capture users' attention, thereby increasing content visibility and attractiveness. Second, adding text to images can provide additional cues and contextual information, further enhancing message clarity and comprehension (23). Lastly, feature complexity reflects the richness of elements and perceived details in images (24). This complexity enhances the richness of the medium by augmenting the detail and diversity of image information. In summary, we propose three distinct visual content strategies to engage audiences on social media: 1. using more images; 2. adding text to images; 3. using images with high feature complexity. These three visual strategies offer more informative content, which, in turn, reduces user uncertainty and fosters audience engagement.

H2: There are significant predictions of audience engagement by visual message content (1. using more images; 2. adding text to images; 3. using images with high feature complexity).

In terms of textual message style, the dimensions include relative equality and responsiveness. Therefore, this study combines social presence theory, which emphasizes the warmth and sociability that media can provide to users (25). To measure social presence, Rourke identified three behavioral indicators: affective, interactive, and cohesive strategy, through a content analysis of online discussions (26). First, the affective strategy reflects socioemotional communication by users who display their feelings and openness. Such behaviors indicate that people strategically use digital platforms to convey a sense of warmth, intimacy, and connectedness (27). Second, the interactive strategy captures behaviors such as asking questions, quoting others, and complimenting others to develop meaningful social interactions (28). The interactive strategy signals the collaborative intentions of communicators by engaging in meaningful social interactions (29). Finally, communicators use a cohesive strategy to foster a sense of group commitment. People often use inclusive pronouns, phatic communication, and refer to the public by name to signal a sense of online community.

Our study also draws on these three strategies of social presence—namely, 1. affective strategy; 2. interactive strategy; and 3. cohesive strategy—to examine how health communicators can utilize social presence strategy to engage audiences.

H3: There are significant predictions of audience engagement by textual message style (1. affective strategy; 2. interactive strategy; and 3. cohesive strategy).

In terms of visual message style, visuals have the ability to elicit emotions, which in turn affect perception, attitude, and behavior (30). Visual effects can convey complex information in an easily understandable way and are more persuasive than text alone (31), thus enabling users to have a more immersive experience with the content. Studies have found that the use of visual effects positively impacts audience engagement (32, 33). Firstly, regarding basic features, we predicted that brightness, contrast, and saturation affect an image's popularity on social media. Higher saturation, contrast, and brightness are often associated with emotional arousal, leading to stronger engagement and promoting information diffusion. Secondly, concerning color features, studies have suggested that the use of warm colors in images is more advantageous (34). Warm colors cause emotional arousal and enhance viral communication on social media. In summary, to study the visual elements of health messages, we propose the following visual features: basic features and color features.

H4: There are significant predictions of audience engagement by visual message style (1. basic features; 2. color features).

## 3 Methods

### 3.1 Data and study sample

Xiaohongshu, also known as Little Red Book, is a content-sharing site where users post text, images, and short videos. Often referred to as China's answer to Instagram, Xiaohongshu is a highly popular social media platform. By the end of December 2023, Xiaohongshu had over 300 million monthly users, and this number continues to grow rapidly. The most active user group is dominated by individuals born after 1990. Xiaohongshu generates three billion views per day, with 70% stemming from user-generated content (35).

Xiaohongshu's unique feature is its heavy reliance on images and short video clips as the primary forms of content creation and sharing (36). As a platform where users share personal experiences in a highly detailed and personalized manner, Xiaohongshu is ideal for gathering in-depth information about users' posts related to their health and disease experiences.

This study conducted a quantitative content analysis of posts published by Xiaohongshu users, focusing on posts about disease experiences from patients and their families. To obtain samples, we first searched using keywords related to emerging infectious diseases (e.g., “COVID-19,” “influenza,” “pneumonia”). Consistent with previous studies, the posts were retrieved using a real-time search algorithm for these keywords (37).

We used Python to collect all content from October 1, 2023, to February 1, 2024, gathering a total of 3,520 data points. We then screened the posts to ensure that the posts contained health messages relevant to individuals' disease experiences. After excluding duplicate and irrelevant posts, we selected 1,824 valid data points as the research sample.

### 3.2 Measurements of variables

We conducted a content analysis of the features of these posts, including textual and visual messages, and developed a coding scheme, as shown in Table 1.

**Table 1:** Operationalization of variables.

**Variables**	**Strategies**	**Sub-types**	**Operationalization**	**Variable type**	**Data source**
Textual message content	Providing event-based information on disease processes	Detection and diagnosis	Description of symptoms and disease detection.	Categorical	Manual
		Treatment	Medication (including precautions, dosage, efficacy, etc.), treatment plans, and non-pharmacological therapies.	Categorical	Manual
		Management	Control of key health indicators, diet, lifestyle habits, and sleep routines.	Categorical	Manual
		Outcomes	Positive results or negative results.	Categorical	Manual
		Advice	Providing informational support and recommendations to others.	Categorical	Manual
		Others	Inquiries regarding health insurance, medical expenses, related healthcare policies, as well as content on respiratory disease education and scientific research.	Categorical	Manual
	Providing information-focused language cues	Post length	Total number of words.	Continuous	LIWC
		Numbers	Number of numbers divided by post length.	Continuous	LIWC
		Cognitive words	Number of cognitive processing words divided by post length.	Continuous	LIWC
	Providing source credibility cues	Official	Government, local/international public health institutions, etc.	Dummy	Manual
		Medical experts	Healthcare professionals with medical expertise, such as doctors, nurses, pharmacists, and other specialists.	Dummy	Manual
Textual message style	Affective strategy	Positive emotions	Sentimental valuation is calculated as positive value.	Categorical	NRC
		Negative emotions	Sentimental valuation is calculated as negative value.	Categorical	NRC
		Emojis	Number of emojis divided by post length.	Continuous	LIWC
	Cohesive strategy	First-person plural pronouns	Number of first-person plural pronouns divided by post length.	Continuous	LIWC
		Informal expressions	Number of informal expressions divided by post length.	Continuous	LIWC
		Caring words	Number of caring words divided by post length.	Continuous	LIWC
	Interactive strategy	Hashtags	Number of hashtags.	Continuous	LIWC
		At-mentions	Number of at-mentions.	Continuous	LIWC
		Question marks	Number of question marks divided by post length.	Continuous	LIWC
Visual message content	Media richness	Adding text	Add text to the image.	Dummy	OpenCV
		Number of images	Number of images.	Continuous	Xiaohongshu
		Visual complexity	The degree of complexity in the textures, patterns, and details of an image.	Continuous	OpenCV
Visual message style	Basic features	Brightness	The overall level of lightness in an image.	Continuous	OpenCV
		Contrast	The degree of difference between colors and brightness in an image.	Continuous	OpenCV
		Saturation	The intensity or vividness of the colors in an image.	Continuous	OpenCV
		Sharpness	The clarity of patterns and details within an image.	Continuous	OpenCV
	Color features	Color variety	The number of different colors present in an image.	Continuous	OpenCV
		Dominant color tone	The most frequently occurring color or hue in an image, categorized into warm, cool and neutral colors.	Categorical	OpenCV
Control variables	Communicator characteristics	Following count	The number of accounts a user follows on a social media platform.	Continuous	Xiaohongshu
		Follower count	The number of users who follow the account on a social media platform.	Continuous	Xiaohongshu
		Favorites and likes	Number of favorites and likes on the homepage.	Continuous	Xiaohongshu
Dependent variable	Audience engagement	Comments	Number of comments received.	Continuous	Xiaohongshu
		Favorites	Number of favorites received.	Continuous	Xiaohongshu
		Likes	Number of likes received.	Continuous	Xiaohongshu
		Shares	Number of shares received.	Continuous	Xiaohongshu

#### 3.2.1 Measurement of independent variables

In terms of text analysis, two main independent variables were covered: message content and message style. The first variable examined textual content strategies, coded into the following dimensions: (1) providing event-based information on disease processes, (2) providing information-focused language cues, and (3) providing source credibility cues.

For event-based information on disease processes, our coding scheme was adapted from the study by Ma et al. (3), which developed a comprehensive framework for disease event processes, including prevention, diagnosis, detection, treatment, and survivorship. However, as this framework was developed in a cancer context, it is not fully applicable to our study. Thus, combining the common-sense model of emerging infectious diseases and illness self-regulation (18), this study ultimately coded disease process events as diagnosis and detection, treatment, management, outcomes, advice, and others.

Information-focused language cues were operationalized based on three dimensions: post length, use of numbers, and use of cognitive words. First, post length refers to the number of words in the text, with a greater number of words potentially indicating more useful information. Second, the use of numbers provides detailed data, accurately describing information in an objective manner and offering highly verifiable facts (38). In health communication, citing statistical data about diseases has been shown to increase engagement (39). Lastly, the use of cognitive words reflects the depth of the author's thinking (14), particularly in the “cognitive processing” category, indicating thought on causal relationships, insight, discernment, and certainty.

Source credibility cues were operationalized based on two dimensions: discourse from medical experts and official discourse. Information from the government, medical experts, and health institutions is considered credible (40). In times of crisis, using reliable sources such as medical experts and public health institutions helps the public share health information (41).

The second variable involves textual style strategies, coded into three sub-dimensions: affective strategy, interactive strategy, and cohesive strategy.

Affective strategy was operationalized in three dimensions: positive emotions, negative emotions, and use of emojis. We measured the emotional valence of each post, involving positive and negative emotions in personal information experiences (42). Additionally, we examined the use of emojis in the posts. Emojis are effective tools for conveying emotions and can enhance the intimacy between communicators (43).

Interactive strategy was operationalized in three dimensions: use of question marks, at-mentions, and hashtags. Using more question marks in posts encourages more audience engagement (14), which helps share messages in an interactive way. Furthermore, we adopted variables from previous literature (44, 45), and modified the use of at-mentions and hashtags into the coding table to fit the current study. Using the at-mentions allows interaction with other higher-traffic accounts, increasing the likelihood of gaining followers. Setting topics with hashtags facilitates targeted interactions, generating more discussions.

Cohesive strategy was operationalized in three dimensions: use of informal expressions, caring words, and first-person plural pronouns in the posts. The use of informal expressions is based on the LIWC lexicon's classification, including categories such as assent, fillers, and filled pauses. The use of informal expressions reflects the closeness of the relationship (46). Using caring words not only expresses concern and support for others but also indicates fondness and care. First-person plural pronouns refer to the frequency of using words like “we” and “our” in the posts. Studies have found that using the first-person plural can promote a sense of group identity and belonging, triggering collective action (47).

In terms of visual coding, we primarily examined two dimensions: content features and stylistic features.

Content features aim to measure media richness, which refers to the potential information load of a communication medium, emphasizing its capability to facilitate shared meaning (23). We selected three quantifiable indicators: the number of images, presence or absence of text, and visual complexity.

Stylistic features were measured in terms of color features and basic features. Color is an important attribute of images, conveying different levels of visual information through various color schemes. This study selected two indicators: color diversity and the dominant color tone of the image. The dominant color tone indicates the most frequently appearing color or hue in an image, divided into three categories: cool colors, warm colors, and neutral colors. Finally, in terms of basic features, the study selected four quantifiable metrics: brightness, contrast, saturation, and sharpness.

#### 3.2.2 Measurement of dependent variables

Consistent with previous research (48), we performed natural logarithmic transformations on the four dependent variables: Ln(likes + 1), Ln(favorites + 1), Ln(comments + 1), and Ln(shares + 1). This approach addresses the high skewness and positive skewness in the data distribution. Also, we added 1 to avoid having a zero base.

Audience engagement was measured by the number of likes, favorites, comments, and shares. This measurement reveals the relationship between different levels of engagement and communication strategies. After testing for reliability and validity, all variables were included in the model. We used SPSS for multiple linear regression analysis.

#### 3.2.3 Measurement of control variables

We used communicator characteristics as control variables. These characteristics include the following count and follower count, as well as the number of favorites and likes on the homepage.

### 3.3 Coding scheme and reliability test

The coding scheme combined machine learning (specifically, natural language processing) with manual coding to ensure reliability and validity.

For the text portion, we used automated language processing software Linguistic Inquiry and Word Count (LIWC) program, which analyzes text by matching words with predefined categories to calculate the frequency and percentage of certain language use (14). Additionally, we supplemented our database with the NRC Lexicon for emotion valence coding. The NRC Emotion Lexicon automatically assigns emotional valence (positive, negative, or neutral) to words based on an emotion lexicon and related intensity scores (49).

For image coding, we used OpenCV, a popular open-source computer vision library with hundreds of algorithms. Researchers were able to analyze various image data features, such as color, brightness, and image richness, and detect and recognize image elements. If a post contained multiple images, we analyzed only the first cover image.

Other variables were manually coded. We employed two graduate students for the coding task. They underwent a 2-h training session to establish coding norms. To check for inter-coder reliability, they randomly and independently coded 10% of the samples. The Kappa values exceeded 0.8, indicating sufficiently high and acceptable reliability of the manual coding.

## 4 Results

All analyses were conducted using SPSS version 29.0. Table 2 presents multiple regression analyses, which were employed to predict how health communication strategies are associated with four types of audience engagement. All variance inflation factor (VIF) values were < 5, indicating no multicollinearity.

**Table 2:** Results of linear regression analysis on audience engagement.

**Variable**	**Comments**	**Favorites**	**Likes**	**Shares**
**Message content**				
**Textual message content**				
**Providing event-based information on disease processes (reference: detection and diagnosis)**				
Treatment	0.045^*^	0.062^**^	0.056^**^	0.056^*^
Management	−0.060^**^	−0.023	−0.011	−0.049^*^
Outcomes	0.008	0.013	0.016	0.02
Advice	−0.039	0.091^***^	0.076^**^	0.066^**^
Others	−0.053^*^	−0.001	0.00	−0.057^*^
**Providing information-focused language cues**				
Post length	−0.037	0.065^*^	0.014	0.071
Numbers	0.057^*^	0.029	0.022	0.02
Cognitive words	0.042	0.05	0.059^*^	0.053
**Providing source credibility cues**				
Official	−0.001	0.005	0.013	0.042^*^
Medical experts	0.066^**^	0.033	0.019	0.044^*^
**Visual message content**				
**Media richness**				
Adding text	−0.083^***^	−0.001	−0.062^**^	0.016
Number of images	−0.051^*^	0.047^*^	0.043	0.022
Visual complexity	−0.099^***^	−0.057^*^	−0.055^*^	−0.066^**^
**Message style**				
**Textual message style**				
**Affective strategy**				
Negative emotions (reference: positive emotions)	0.075^**^	−0.026	−0.024	0.005
Emojis	0.032	−0.035	−0.005	−0.029
**Cohesive strategy**				
First-person plural pronouns	0.021	0.019	0.032	0.018
Informal expressions	0.058^*^	−0.033	−0.026	−0.049
Caring words	0.054^*^	0.045^*^	0.053^**^	0.048^*^
**Interactive strategy**				
Hashtags	−0.042	0.074^*^	0.048	0.04
At-mentions	0.01	0.025	0.024	0.00
Question marks	−0.006	0.015	−0.001	0.051^*^
**Visual message style**				
**Basic features**				
Brightness	−0.101^**^	−0.063^*^	−0.052	−0.057
Contrast	0.045	0.023	0.037	0.041
Saturation	−0.039	−0.027	−0.011	−0.001
Sharpness	−0.017	0.013	0.004	0.026
**Color characteristics**				
Color variety	−0.024	−0.001	−0.027	−0.026
Dominant color (reference: cold colors)				
Neutral colors	0.023	0.024	0.018	0.027
Warm colors	0.031	0.006	0.018^**^	−0.014
**Control variables**				
Following count	−0.014	−0.053^**^	−0.066^***^	−0.049^*^
Follower count	−0.130^**^	−0.186^***^	−0.201^***^	−0.156^***^
Favorites and Likes	0.447^***^	0.567^***^	0.608	0.513^***^
F-value	11.893^***^	22.095^***^	20.993^***^	18.857^***^
R^2^	0.18	0.289	0.279	0.258
Adjusted R^2^	0.165	0.276	0.266	0.244

Dependent variables and control variables were log-transformed before entering into the model. ^*^p < 0.05; ^**^p < 0.01; ^***^p < 0.001.

For H1, in terms of providing event-based information on disease processes, this study found that posts using uncertainty reduction strategies had an impact on public engagement. Posts that mentioned treatment had a positive effect on comments (β = 0.045, *p* < 0.05), favorites (β = 0.062, *p* < 0.01), likes (β = 0.056, *p* < 0.01), and shares (β = 0.056, *p* < 0.05). Posts that mentioned advice positively predicted an elevated number of favorites (β = 0.091, *p* < 0.001), likes (β = 0.076, *p* < 0.01), and shares (β = 0.066, *p* < 0.01). Conversely, posts that mentioned management were negatively predicted to decrease both comments (β = −0.060, *p* < 0.01) and shares (β = −0.049, *p* < 0.05), highlighting their potential detrimental role in fostering public discussions. Others had a negative effect on comments (β = −0.053, *p* < 0.05) and shares (β = −0.057, *p* < 0.05). However, posts that mentioned outcomes indicated no significant effect on audience engagement. Regarding information-focused language cues, post length was positively associated with an increased number of favorites (β = 0.065, *p* < 0.05). The use of numbers was also found to positively predict a higher number of comments (β = 0.057, *p* < 0.05). Using cognitive words had a positive effect on likes (β = 0.059, *p* < 0.05). When providing credibility cues for sources, mentioning official sources had a significantly positive impact on shares (β = 0.042, *p* < 0.05). Similarly, mentioning a physician as a source had a significantly positive effect not only on comments (β = 0.066, *p* < 0.01) but also on shares (β = 0.044, *p* < 0.05).

For H2, the number of images had mixed effects: negatively affecting comments (β = −0.051, *p* < 0.05) and positively affecting favorites (β = 0.047, *p* < 0.05). However, no significant effect on likes was observed. Adding text had a significant negative impact on both comments (β = −0.083, *p* < 0.001) and likes (β = −0.062, *p* < 0.01), but no significant effect on favorites was found. Additionally, visual complexity negatively predicted comments (β = −0.099, *p* < 0.001), favorites (β = −0.057, *p* < 0.05), likes (β = −0.055, *p* < 0.05), and shares (β = −0.066, *p* < 0.01).

For H3, we found that the message style used to demonstrate social presence was highly effective in cultivating audience engagement. In terms of affective strategies, posts containing negative emotions had a significant positive effect on comments (β = 0.075, *p* < 0.01). Regarding interactive strategies, using hashtags was positively associated with an increased number of favorites (β = 0.074, *p* < 0.05). In terms of cohesive strategies, the use of informal expressions was associated with a higher number of comments (β = 0.058, *p* < 0.05). Additionally, the use of caring words had a significant positive effect on comments (β = 0.054, *p* < 0.05), favorites (β = 0.045, *p* < 0.05), likes (β = 0.053, *p* < 0.01), and shares (β = 0.048, *p* < 0.05). However, we have not yet observed a significant effect of other variables, such as the use of emojis, first-person plural pronouns, at-mentions and question marks, on engagement.

For H4, regarding basic features, brightness negatively predicted both comments (β = −0.101, *p* < 0.01) and favorites (β = −0.063, *p* < 0.05). No significant effects of other basic visual features, such as contrast, saturation, and clarity, on engagement were found. As for color features, the use of warm colors was associated with a higher number of likes (β = 0.018, *p* < 0.01). No other color features were found to significantly affect engagement.

Therefore, there was sufficient evidence to partially support H1, H2, H3, and H4.

## 5 Discussion

### 5.1 Summary of major findings

The COVID-19 pandemic has been a pivotal event that reshaped the way health information is communicated, both on social media and in general public discourse. Before the pandemic, individuals often relied on individualistic coping strategies to manage health-related concerns. However, the uncertainty and collective challenges brought about by the pandemic have shifted this dynamic. People began seeking more collective communication approaches, looking for social support and clarification. This shift in communication strategies, from individual to collective, became even more apparent in the aftermath of the pandemic. In terms of message content, individuals have moved toward sharing and seeking information in more public narratives, which have been essential in reducing uncertainty in health risk communication. By providing clear, reliable, and rational appeals, uncertainty can be reduced, thus increasing audience engagement. The uncertainty associated with infections and the need for collective coping have led to the prevalence of public narratives and expressions; this has created a unique social environment on social media, offering an additional means to respond to health-related public emergencies (50). The advantage of online health communities lies in their ability to shift risk communication from monologue to dialogue. When strategies are appropriately used within personal health experience content, they can enhance public participation, which is crucial for health promotion. At the same time, messages shared must include rational appeals, addressing logical and evidence-based aspects of health risks to foster trust and facilitate informed decision-making.

In terms of textual content, previous studies have identified that medical or scientific uncertainty encompasses questions regarding diagnosis, prognosis, and treatment options (51). Regarding the provision of event-based information on disease processes, many posts incorporate details about events occurring at various stages of the disease. These details can predict audience engagement, as they represent the type of information sought and aid in forming a mental representation of the disease (52). Information pertaining to detailed treatment processes (e.g., medications, treatment plans) predicts higher numbers of comments, favorites, and likes. Similarly, posts that offer advice also predict a higher number of likes and favorites. In contrast, content related to management processes, such as indicator control, diet, routine, health insurance, and medical expenses, negatively correlates with public engagement. This is because respiratory infections are acute diseases, and audience engagement typically hinges on the usefulness and relevance of the information. Users tend to be more intrigued by finding swift recovery methods to mitigate uncertainty and focus on immediate solutions, rather than long-term or less pressing post-management processes.

When messages provide information-focused language cues, longer post lengths positively predict the number of favorites, as posts of greater length typically offer more information, albeit with varying quality. Users tend to favor content that enhances their understanding, increasing the likelihood that longer posts will attract more favorites. Posts that include numerical data, such as medication dosages, treatment durations, and disease statistics, are more likely to be perceived as credible and objective, thereby increasing user engagement (38). Accurate and verifiable data are critical for building trust, as users are more inclined to engage with content they believe is backed by scientific evidence. Cognitive words are frequently used to explain events with a high degree of uncertainty, reflecting the writer's deeper thinking and understanding of the experience (53). Using more cognitive processing words helps individuals better comprehend the writer's experiences, ultimately leading to a greater likelihood of favorable interactions.

In terms of providing source credibility cues, there is an objective need for medical experts and official sources to assist the public in bridging the information gap, particularly when they are unaware of emerging health risks. Explicit references to medical experts' discourse can encourage more dialogic communication and interaction. As found in previous studies, the government, medical experts, and government health agencies are trusted sources of health information (54). Thus, mentioning medical experts' discourse positively predicts the number of comments. Explicitly mentioning official sources can lead to greater diffusion of information and is positively correlated with the number of shares.

In relation to visual characteristics, contrary to the positive effects of visual elements on health outcomes demonstrated in prior work (32, 55), our findings reveal that high visual complexity significantly reduces audience engagement. From a “less is more” perspective, visually simple stimuli may be processed more effortlessly, fostering more favorable perceptions (34, 55). According to media richness theory, higher media richness does not always equate to better outcomes; rather, it depends on the specific requirements of the organizational task (23). In essence, optimal results are achieved when media richness aligns with the objectives. Particularly during a health crisis, when users' social media engagement primarily revolves around information needs, excessive visual complexity can impose a visual burden and distress upon users seeking health information. This, in turn, can divert attention, heighten uncertainty, and decrease the likelihood of shares, likes, and comments.

In addition to message content, communicators can also use message style as strategies to activate emotional appeals, evoking understanding and empathy from others toward their experiences. This emotional appeal makes the voice of patients' personal experiences more relatable and more likely to capture the audience's attention and reflection on post-pandemic acute infectious disease issues, thereby stimulating user engagement. Patients seeking health information expect warm, pleasant, and relaxed interactions. Online health information exchanges primarily rely on text and images on electronic screens. The use of text strategies that convey social presence and warm-toned visual strategies helps bridge the gap between communicators and audiences in virtual spaces. Therefore, this communication approach not only satisfies users' emotional needs and improves information quality, but also aids in cognitive processing and fosters interpersonal and emotional connections.

As for textual message style, specifically in terms of affective strategy, previous research has found that negative emotions enhance the usefulness of online reviews (56). Our study also revealed that posts containing negative emotions attract more comments. This may be due to the fact that negative emotions are prevalent on Xiaohongshu when discussing the relatively specific topic of illness, and a higher degree of negative emotions is more likely to elicit others' attention and comments.

Regarding cohesive strategy, the warmth of communication may also play a role in the three different types of engagement. Using caring words fosters warmth and closeness in interpersonal interactions (57). Using informal expressions positively predicts the number of comments because it presents a relaxed and casual atmosphere in which other users are more likely to participate in the discussion. It has been shown that the use of informal expressions in social media communication increases perceived warmth (58).

In terms of interactive strategy, using hashtags to set topics allows post content to receive targeted and contextual interactions, allowing users to find relevant shares on a particular topic or event (44), which promotes more audience engagement. Posts with question marks tend to be shared by more users (59), because structuring interactive messages in the form of posted questions encourages online users to participate more actively in communication.

As for visual message style, our study shows that warm colors play an important role in predicting the number of likes, and it is more advantageous to use warm colors in images. Our findings are consistent with existing research (34), further confirming that the use of warm colors causes emotional arousal and increases the number of likes on social media.

### 5.2 Practical implications

The findings of this research have several important implications. Firstly, we have developed a comprehensive social media information framework that systematically examines effective health message strategies, encompassing both message content and message style. This framework not only provides insights into how health communicators can leverage social media platforms to share personal health experiences and stories, but also highlights how these strategies can attract greater audience engagement by making health messages more relatable and impactful. Specifically, the use of personal health experiences informs more persuasive messaging, increasing the likelihood of resonating with the audience on a deeper level. Moreover, this study offers valuable guidance for governments and health agencies aiming to improve their social media engagement strategies. By better understanding how different communication tactics influence audience interaction, our findings support more effective public health guidance, particularly in the context of acute infectious disease outbreaks. For example, we suggest employing uncertainty-reducing strategies that provide clear, credible information to alleviate audience concerns. Additionally, social presence strategies should be employed to actively foster engagement and create dialogic communication around health issues. Finally, our research underscores the importance of visual strategies, advocating for the strategic use of visual elements to enhance engagement and ensure messages are both informative and emotionally resonant. These insights collectively offer practical guidance for designing health promotion campaigns that are not only informative but also foster meaningful interaction and connection with the audience.

### 5.3 Limitations

There are several limitations to this study. First, the present study relied on Xiaohongshu to collect data. Future studies could investigate the impact of health message strategies on audience engagement across different platforms, such as TikTok and Sina Weibo. Second, although quantitative indicators such as likes, favorites, and comments have become standards to measure audience engagement, some studies suggest that more appropriate indicators beyond simply number of likes are needed. For example, audience engagement can be divided into cognitive, emotional, and participatory dimensions (60). Thirdly, visual elements constitute a distinctive aspect of health communication. While visual effects have the potential to significantly enhance audience engagement, they also introduce the risk of visual deception, where misleading or false imagery can distort health-related messages. Therefore, future research should aim to develop a comprehensive approach that enables a more rigorous examination of visual components. This approach would not only help identify and mitigate instances of visual deception, but also explore the mechanisms through which platforms can self-regulate to ensure the accuracy and authenticity of visual content used in health communication.

## 6 Conclusion

This study analyzed health communication strategies on the Xiaohongshu platform, focusing on how they influence audience engagement during emerging infectious disease outbreaks post-COVID-19. We developed a model to examine the effects of textual and visual elements on user engagement. Our analysis of 1,824 posts revealed that informational content, particularly event-based details and information-focused language cues, positively impacted engagement by addressing rational needs. In contrast, overly complex visuals hindered engagement, suggesting that simplicity in visual communication is more effective. Regarding message style, affective, interactive, and cohesive strategies, along with the use of warm colors, were found to enhance engagement. These findings underscore the importance of clear, credible information, emotional resonance, and social connection in engaging audiences during health crises. The study offers practical insights for health communicators, recommending strategies that reduce uncertainty, foster social presence, and use visuals strategically to boost engagement. These approaches can improve public health communication and encourage collective action, providing valuable guidance for governments and health agencies to refine their social media engagement strategies in future health emergencies.

## Data Availability

The raw data supporting the conclusions of this article will be made available by the authors, without undue reservation.
